# Unilateral Absence of the Left Pulmonary Artery With an Associated Vascular Anomaly in Adulthood

**DOI:** 10.7759/cureus.527

**Published:** 2016-03-10

**Authors:** Miguel Flores, Haley Letter, Edward Derrick, Ibrahim Koury

**Affiliations:** 1 Diagnostic Radiology, Florida Hospital-Orlando

**Keywords:** uapa, pulmonary artery agenesis, cardiovascular anomaly, congenital

## Abstract

Left-sided pulmonary artery agenesis is a rare malformation that commonly requires childhood intervention secondary to associated congenital cardiovascular anomalies. We present an uncommon case of left-sided agenesis with an associated right-sided aortic arch and significant hypoplasia of the ipsilateral lung. Additionally, there is radiographic evidence of emphysema and pulmonary artery hypertension. Pulmonary artery agenesis is not a common entity, but should be considered in adult patients presenting with recurrent pneumonias and radiographic evidence suggestive of pulmonary hypoplasia. A prompt diagnosis is beneficial for affected individuals who may be candidates for a revascularization procedure or embolization of collaterals. Earlier diagnosis also allows for proper management and follow-up care, considering pulmonary artery hypertension is a severe complication of pulmonary artery agenesis.

## Introduction

Unilateral absence of pulmonary artery (UAPA) is a rare congenital malformation that may develop in isolation or in association with congenital cardiovascular anomalies [1-4]. In cases of isolated UAPA, incidence has been reported to range from 1 in 200,000 to 1 in 300,000 [3]. Agenesis of the right pulmonary artery has a greater incidence than agenesis of the left. However, left-sided agenesis is more likely to develop with a concurrent congenital cardiovascular malformation, thus leading to patients presenting at a younger age [2,5]. Associated cardiovascular anomalies may include Tetralogy of Fallot (TOF), atrial septal defect, coarctation of the aorta, a right-sided aortic arch, truncus arteriosus, and pulmonary atresia [3]. The affected pulmonary artery is most commonly opposite to the aortic arch. This explains why the more prevalent right UAPA occurs with a normal left-sided arch [5].

UAPA is believed to result from the involution of the proximal sixth aortic arch, thereby forgoing expected embryological fusion with the pulmonary trunk [3,4]. Most common symptoms experienced include recurrent pulmonary infections, hemoptysis, and shortness of breath upon exertion [1-4]. Informed consent from the patient was not required for this study.

## Case presentation

A 69-year-old female with a greater than 100-pack-year smoking history and known emphysema presented to a pulmonologist for worsening shortness of breath. The patient reported a remote history of pneuomonia, increased shortness of breath with mild physical activity, audible wheezing, and chest tightness. She denied lower extremity swelling, paroxysmal nocturnal dyspnea, or palpitations. There was no history of cardiac disease or cardiac surgery.

The patient underwent initial chest imaging at an outside facility and the imaging was not available at the time of presentation at our institution. A chest radiograph revealed hyperinflation of the right lung with leftward shift of the mediastinum. The interpreting radiologist noted left lower lobe volume loss and recommended further evaluation to exclude an obstructing endobronchial mass. 

**Figure 1:**
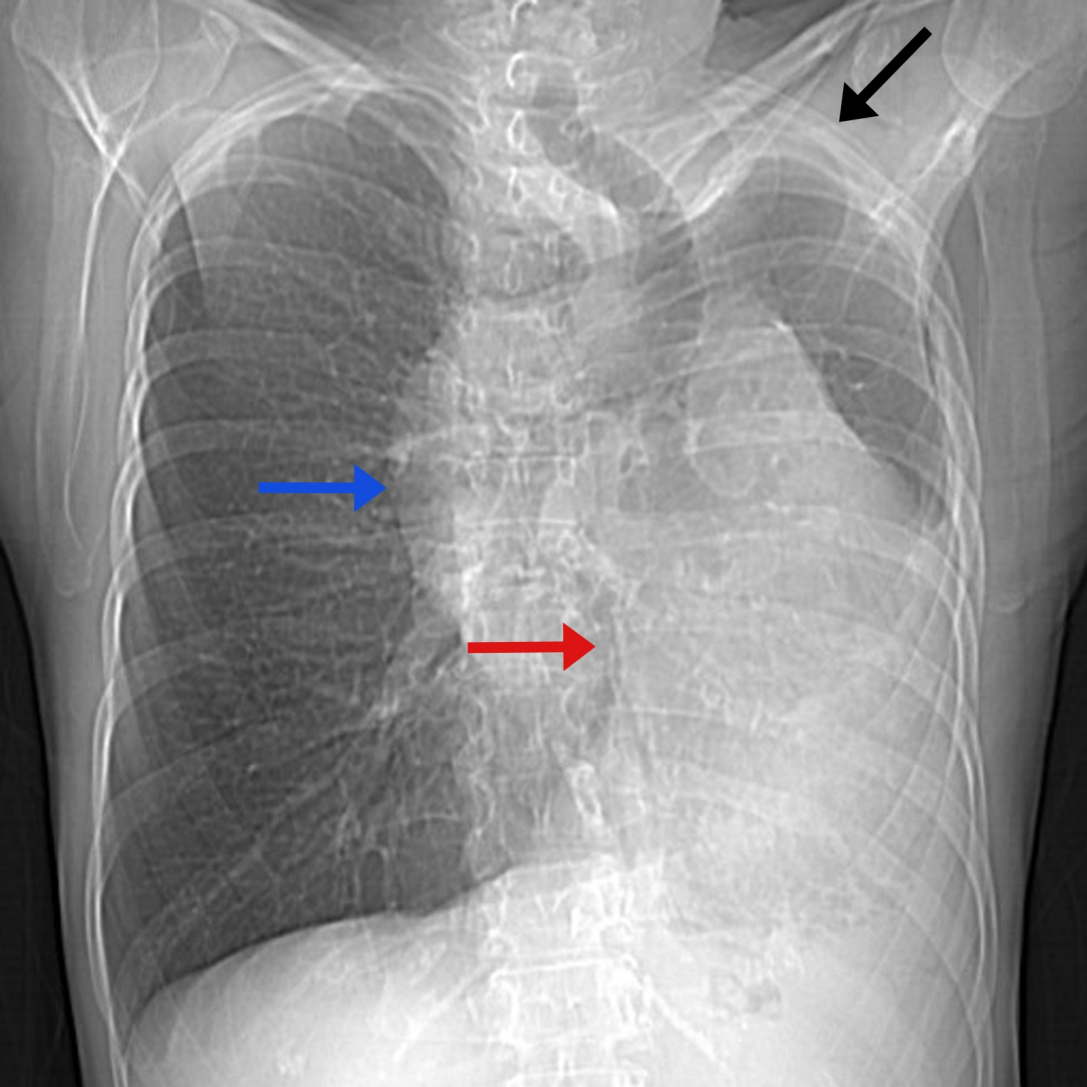
CT chest, scout image. There is leftward shift of the cardiomediastinal silhouette (red arrow) with hyperinflation of the right lung; no evidence of left lobe collapse. The left hemithorax is asymmetrically smaller when compared to the right (black arrow). There is central left-sided bronchiectasis with opacification of the left lower lung field. Incidental note is made of a right-sided aortic arch (blue arrow).

**Figure 2:**
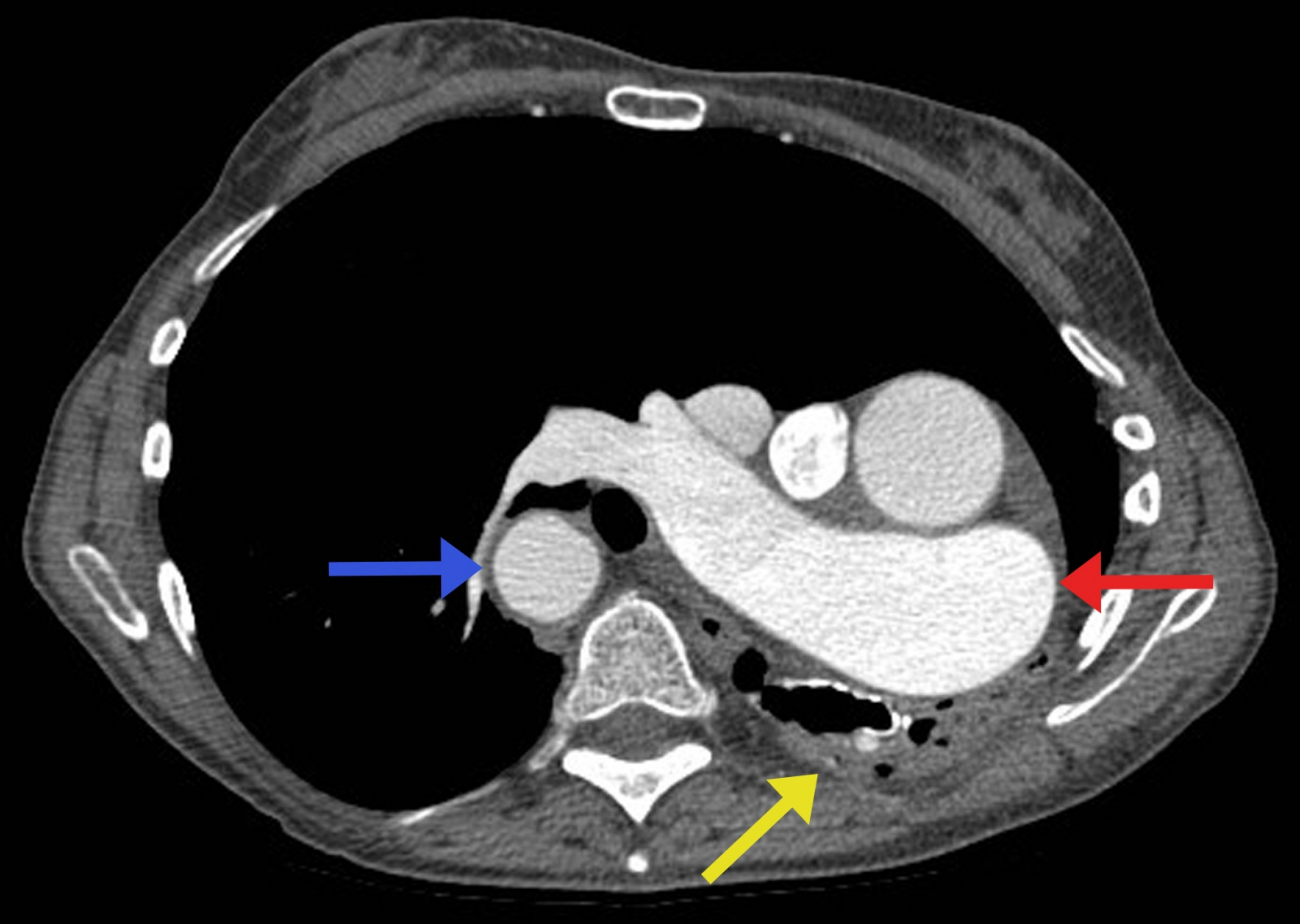
Contrast-enhanced CT chest, axial image. Pulmonary trunk emerges from the right ventricular outflow track and entirely feeds deoxygenated blood into an enlarged right pulmonary artery (red arrow). The left lung is almost entirely hypoplastic with a few dilated bronchi/bronchioles and small caliber vessels (yellow arrow). Incidental note is made of a right-sided aortic arch (blue arrow).

**Figure 3:**
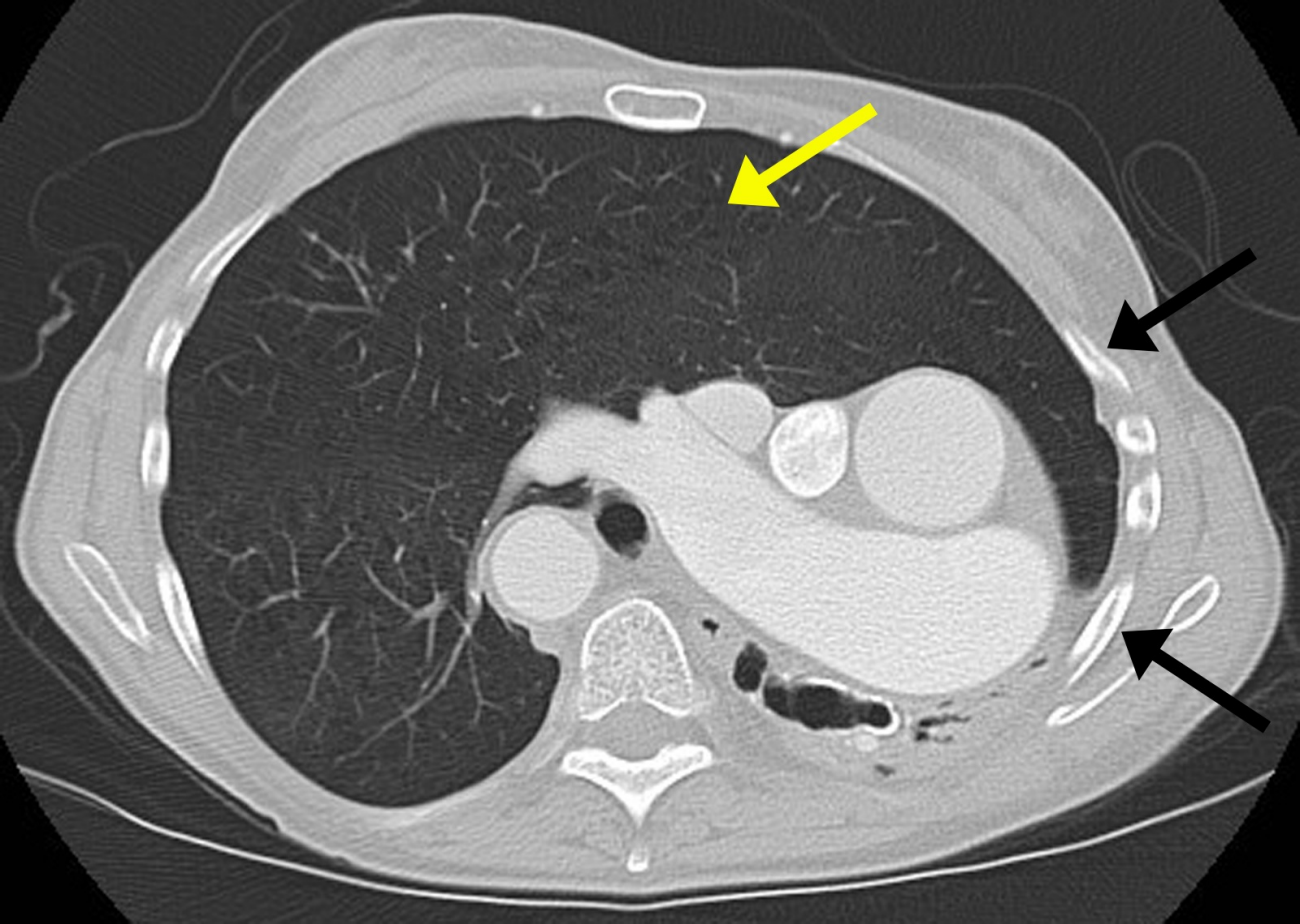
Contrast-enhanced CT chest, axial image in lung window. There is hyperinflation and expansion of the right lung, almost occupying the entire thoracic cavity (yellow arrow). The left chest is asymmetrically smaller when compared to the right (black arrow).

**Figure 4:**
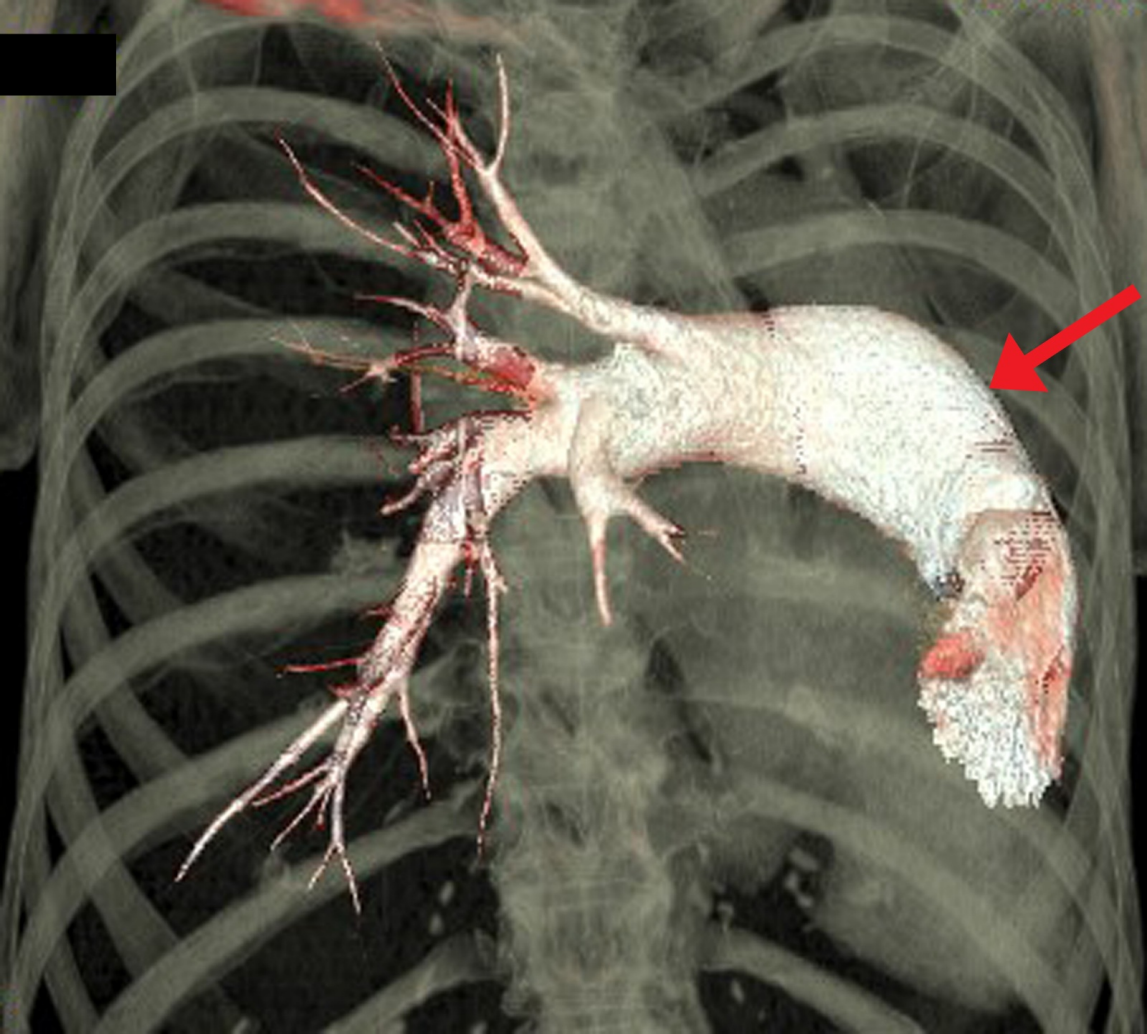
3D volume-rendered reconstruction of the pulmonary trunk and right pulmonary artery. Reconstruction demonstrates a single, dilated right pulmonary artery arising from the pulmonary trunk (red arrow). No left pulmonary artery or hypoplastic remnant visualized.

**Figure 5:**
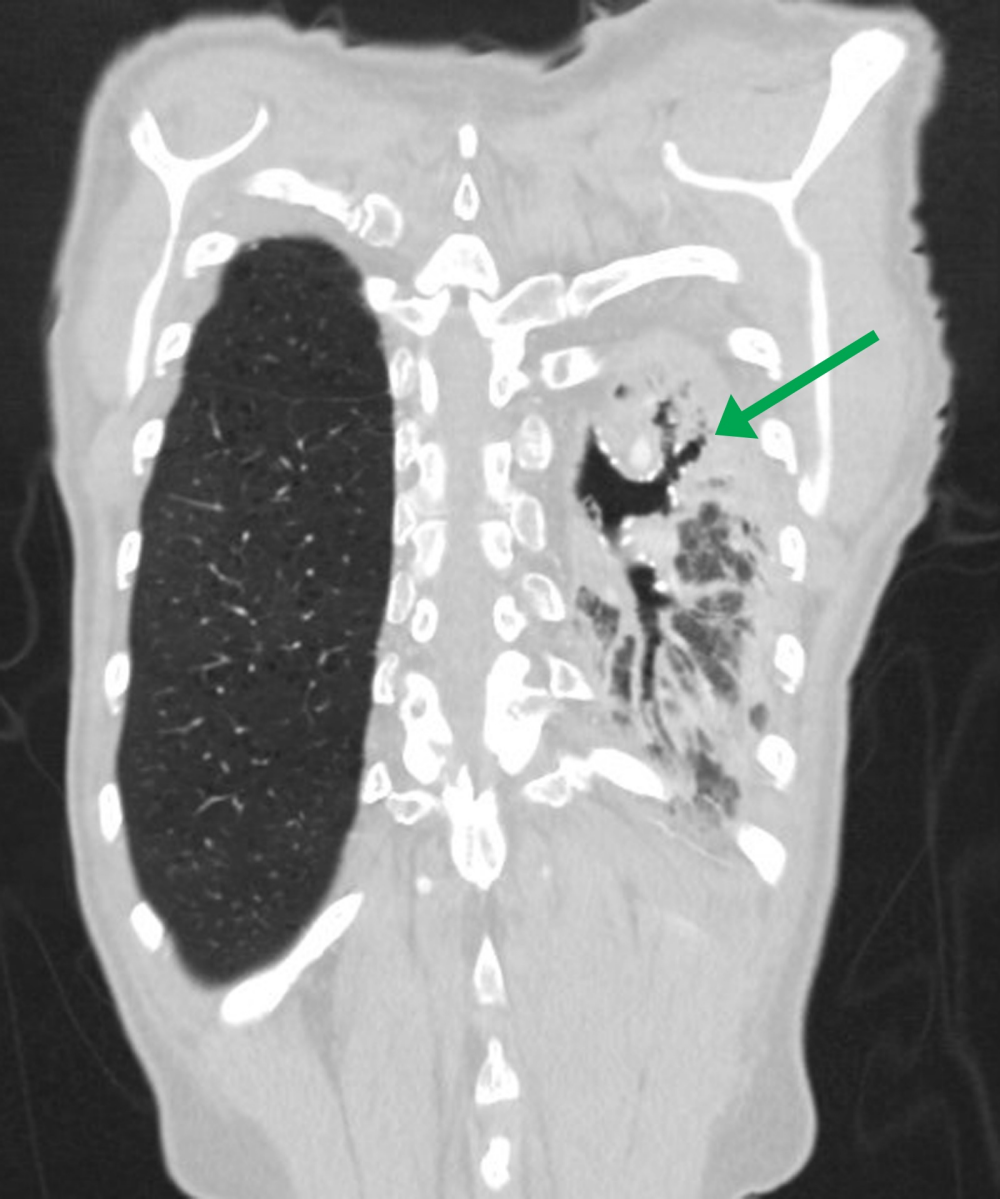
Contrast-enhanced CT chest, coronal reformation in lung window. Bronchiectasis may be seen in cases of pulmonary artery agenesis secondary to recurrent pulmonary infections (green arrow).

Subsequent contrast-enhanced computed tomography (CT) of the chest (Figures 1-5, Video 1) at our institution revealed left-sided pulmonary artery agenesis with an associated right-sided aortic arch and significant hypoplasia of the ipsilateral lung. Hyperinflation and expansion of the remaining right lung with evidence of emphysema and pulmonary artery hypertension were also observed. 

**Video 1:** 3D volume-rendered reconstruction of the pulmonary trunk and right pulmonary artery.

## Discussion

Left-sided agenesis is a rare entity that occurs half as frequently as right-sided agenesis. The development of left-sided agenesis has been described in two different subtypes [3]. Approximately 80% of left-sided agenesis is associated with a congenital cardiovascular malformation necessitating medical attention in early life [3]. A second, though less frequent, subset of those with left-sided agenesis will develop isolated left pulmonary artery agenesis without associated cardiovascular anomaly, thus presenting later on in life. In the case of our patient, left-sided agenesis developed with an associated vascular anomaly, a right-sided aortic arch, which was not clinically significant in younger life.

The typical radiographic findings include: absence of an ipsilateral pulmonary artery shadow with enlargement of the contralateral shadow, volume loss of the affected ipsilateral lung and ipsilateral mediastinal shift with hyperinflation of the contralateral lung. The findings are nonspecific in an adult patient, but may represent bronchial obstruction from mucus plugging or endobronchial malignancy, prior lobectomy, or congenital pulmonary hypoplasia.

This unique case illustrates a rather uncommon adult cause of mediastinal shift with volume loss of the ipsilateral lung. While there are many causes for lung volume loss on chest radiography, malignancy causing endobronchial obstruction must be excluded in an older patient with a smoking history. In this case, the follow-up contrast enhanced CT of the chest revealed a diagnosis of UAPA, an associated vascular anomaly, development of centrilobular emphysema, and pulmonary artery hypertension.

The treatment for UAPA is patient specific and will vary depending on age of presentation, associated cardiovascular anomalies, and symptomatology. The options in young patients include revascularization of the distal affected pulmonary artery. The procedure results in a systemic-to-pulmonary artery shunt, considering intrapulmonary arteries are still formed during embryological development [3,5]. Lobectomy, or selective embolization of systemic arterial supply, may be considered for patients with hemoptysis secondary to bleeding collateral vessels or patients with persistent pulmonary infections involving a specific lobe [3, 5-6]. Although patients who remain undiagnosed until adulthood may remain asymptomatic, development of pulmonary artery hypertension remains a severe UAPA complication [1,2]. Treatment with an endothelin receptor antagonist may be considered [3]. Regular follow-up and observation of pulmonary hemodynamics is recommended [1].

## Conclusions

Left-sided pulmonary artery agenesis is a rare malformation that commonly requires childhood intervention secondary to associated congenital cardiovascular anomalies. Our patient presented and was diagnosed with a known variant, left-sided agenesis with a right-sided aortic arch, which remained undiagnosed until adulthood. Reaching adulthood in this population is uncommon and initial radiographic findings may mimic malignancy. Most importantly, correct diagnosis is essential for appropriate clinical management and follow-up care, considering pulmonary artery hypertension remains a serious complication. 
